# Global Utilization Trends of Direct Acting Antivirals (DAAs) during the COVID-19 Pandemic: A Time Series Analysis

**DOI:** 10.3390/v13071314

**Published:** 2021-07-07

**Authors:** Ahmad Shakeri, Natalia Konstantelos, Cherry Chu, Tony Antoniou, Jordan Feld, Katie J. Suda, Mina Tadrous

**Affiliations:** 1 Women’s College Research Institute, Women’s College Hospital, Toronto, ON M5S 1B2, Canada; ahmad.shakeri@wchospital.ca (A.S.); n.konstantelos@mail.utoronto.ca (N.K.); cherry.chu@wchospital.ca (C.C.); 2 Leslie Dan Faculty of Pharmacy, University of Toronto, Toronto, ON M5S 3M2, Canada; 3 Department of Family and Community Medicine, St. Michael’s Hospital, Toronto, ON M5B 1W8, Canada; tony.antoniou@unityhealth.to; 4 Li Ka Shing Knowledge Institute, St. Michael’s Hospital, Toronto, ON M5B 1W8, Canada; 5 Toronto General Hospital, University Health Network, Toronto, ON M5G 2C4, Canada; jordan.feld@uhn.ca; 6 Center for Health Equity Research and Promotion, VA Pittsburgh Healthcare System, Pittsburgh, PA 15240, USA; ksuda@pitt.edu; 7 Department of Medicine, School of Medicine, University of Pittsburgh, Pittsburgh, PA 15213, USA

**Keywords:** antivirals, hepatitis C, COVID-19, drug policy, time series

## Abstract

The 2019 novel coronavirus (COVID-19) pandemic has placed a significant strain on hepatitis programs and interventions (screening, diagnosis, and treatment) at a critical moment in the context of hepatitis C virus (HCV) elimination. We sought to quantify changes in Direct Acting Antiviral (DAA) utilization among different countries during the pandemic. We conducted a cross-sectional time series analysis between 1 September 2018 and 31 August 2020, using the IQVIA MIDAS database, which contains DAA purchase data for 54 countries. We examined the percent change in DAA units dispensed (e.g., pills and capsules) from March to August 2019 to the same period of time in 2020 across the 54 countries. Interrupted time-series analysis was used to examine the impact of COVID-19 on monthly rates of DAA utilization across each of the major developed economies (G7 nations). Overall, 46 of 54 (85%) jurisdictions experienced a decline in DAA utilization during the pandemic, with an average of −43% (range: −1% in Finland to −93% in Brazil). All high HCV prevalence (HCV prevalence > 2%) countries in the database experienced a decline in utilization, average −49% (range: −17% in Kazakhstan to −90% in Egypt). Across the G7 nations, we also observed a decreased trend in DAA utilization during the early months of the pandemic, with significant declines (*p* < 0.01) for Canada, Germany, the United Kingdom, and the United States of America. The global response to COVID-19 led to a large decrease in DAA utilization globally. Deliberate efforts to counteract the impact of COVID-19 on treatment delivery are needed to support the goal of HCV elimination.

## 1. Introduction

Close to 71 million people are chronically infected with the hepatitis C virus (HCV) worldwide [1]. If left untreated, chronic HCV infection can cause substantial morbidity and mortality, with complications including cirrhosis, end-stage liver disease, and hepatocellular carcinoma [2,3]. Published models that assume absence of treatment suggest that cases of end stage liver disease among HCV-infected people will peak between 2030 and 2035 [4]. Fortunately, treatment options for HCV infection have improved over the past decade with the introduction of direct-acting antiviral (DAA) treatment. DAAs can achieve sustained virologic responses or cure a high (>95%) proportion of patients with favorable safety and tolerance profiles [5]. Sustained virologic response leads to substantial reductions in liver transplantation, hepatocellular carcinoma, and liver-related mortality, prompting the World Health Organization (WHO) to set targets for HCV elimination by 2030 [6,7,8]. However, this goal requires sustained robust screening programs for HCV and global access to DAAs [9].

The 2019 novel coronavirus (COVID-19) pandemic has impacted all stages of the HCV cascade of care and has resulted in reduced access to critical medical services [10]. Early in the pandemic, the World Hepatitis Alliance administered a global survey assessing the effects of the COVID-19 pandemic on viral hepatitis services [11]. Findings included decreased hepatitis testing due to pandemic-related closures or patient avoidance of testing facilities and a lack of hepatitis treatment access due to temporary government-imposed restrictions in routine healthcare [11]. Although modelling studies suggest that some countries could conceivably eliminate HCV by 2030, they did not account for the COVID-19 pandemic, which may jeopardize progress toward elimination [12]. Yet the impact of the COVID-19 pandemic on global DAA utilization is unknown. We sought to quantify changes in DAA utilization among different countries during the COVID-19 pandemic.

## 2. Materials and Methods

### 2.1. Study Design 

To better understand the impact of the COVID-19 pandemic on global DAA utilization, we conducted a cross-sectional time series analysis examining monthly DAA units sold from 1 September 2018 to 31 August 2020. 

### 2.2. Data Source

Data on DAA (Anatomical Therapeutic Chemical code J05AP) sales were obtained from the IQVIA Multinational Integrated Data Analysis (MIDAS) database [13]. The database contains annual figures summarizing pharmacy sales of specific DAAs and combination DAA units in 54 countries/regions (Guatemala, El Salvador, Honduras, Nicaragua, Costa Rica, and Panama are aggregated as Central America, and Benin, Burkina Faso, Cameroon, Côte d’Ivoire, Democratic Republic of the Congo, Gabon, Guinea, Mali, Senegal, and Togo as Francophone West Africa) [13]. DAA units were defined by IQVIA as a single capsule/pill (generic and brand name formulations; DAA drug list: Table S1). IQVIA’s MIDAS databases includes aggregate units sold but lacks relevant clinical (e.g., indication) and demographic information (i.e., age, sex, number of people living with HCV). The prescription data is projected to represent national prescribing levels across all jurisdictions (Global precision index in 2019: 94% to 95%) and is regularly used for research purposes because it estimates prescription volumes from all public and private payers [14,15,16,17]. In addition, the proportion of total pharmacy sales in each country obtained by IQVIA-MIDAS database ranges from 45% (United Arab Emirates) to 100% (23 out of 54 countries analyzed), Table S2.

### 2.3. Statistical Analysis

To study the impact of the COVID-19 pandemic on global DAA utilization, we compared the percent change in DAA units dispensed from March to August 2019 to the same period of time in 2020, across the 54 jurisdictions available in our database. We also stratified our analysis among high prevalence (defined as a HCV prevalence > 2%) countries in our database (Egypt, Kazakhstan, Pakistan, Romania, Taiwan) [1]. In addition, we report the standardized DAA units sold per 100,000 persons between September 2018 and August 2020 across the Group of Seven countries (G7: Canada, France, Germany, Italy, Japan, the United Kingdom, and the United States of America) [18]. We selected the G7 because they reported complete monthly data from September 2018, whereas other countries did not. We obtained monthly population estimates between 2018 and 2020 [19]. These estimates were used to population standardize DAA units sold. 

Finally, we used autoregressive integrated moving average models (ARIMA) to examine the impact of COVID-19 on monthly rates of DAA utilization across each of the G7 countries [20]. ARIMA modelling is a robust method for assessing population-level health intervention impacts, as it can account for underlying trends, autocorrelation, and seasonality and allows for the modelling of various types of interventions [21]. This form of analysis is used to study drug utilization data as they often exhibit seasonality and autocorrelation in their trends. We examined the impact of the early months of the COVID-19 pandemic by including a ramp intervention function to detect a change in trend of monthly DAA utilization after March 2020. *p* value less than 0.05 was considered significant. All analyses were performed using SAS (version 9.4, The SAS Institute).

## 3. Results

**Figure 1:**
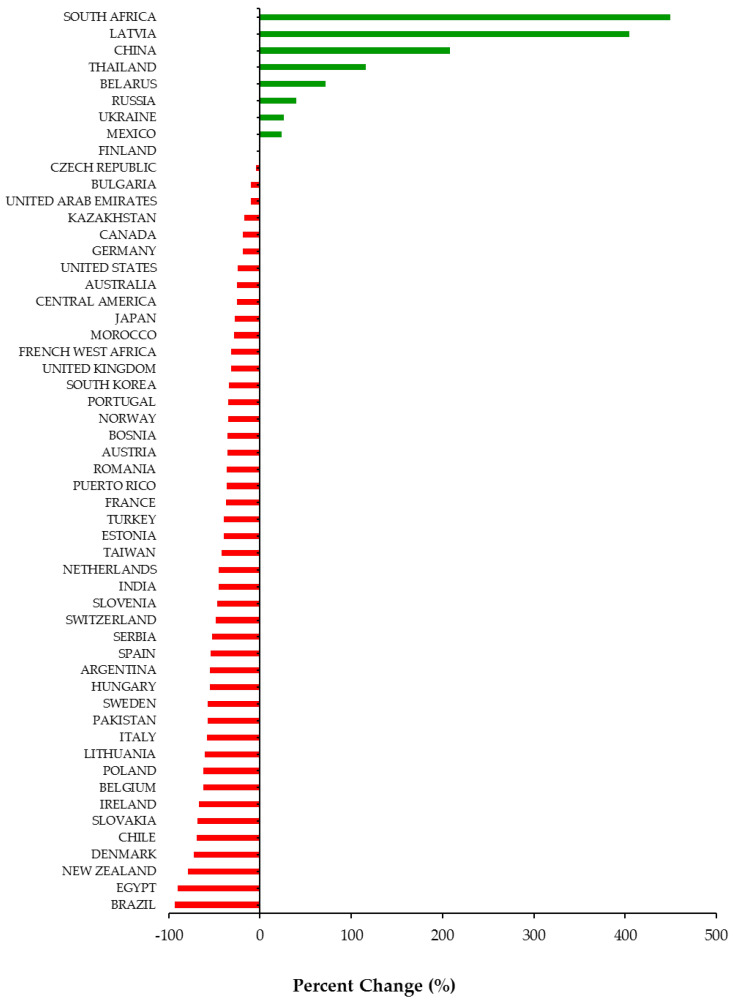
Percent change (%) in utilization of Direct Acting Antivirals by country and region from March to August 2020 compared to March to August 2019.

Overall, 46 out of 54 (85%) jurisdictions available in our database experienced a decline in DAA utilization during the COVID-19 pandemic (Figure 1). Among the 46 jurisdictions, the utilization of DAAs decreased on average by −43% (Standard Deviation (SD = 21)) during the first 6 months of the pandemic (March to August 2020). Decreases in DAA utilization ranged from −1% in Finland to −93% in Brazil. Among the eight countries that experienced a percent increase, the range was between +24% (Mexico) to +449% (South Africa). All countries in our database with a high prevalence of HCV (Egypt, Kazakhstan, Pakistan, Romania, Taiwan) experienced a −49% (SD = 27) average decline in DAA utilization. Decreases in DAA utilization in high prevalence countries ranged from −17% in Kazakhstan to −90% in Egypt. 

**Figure 2:**
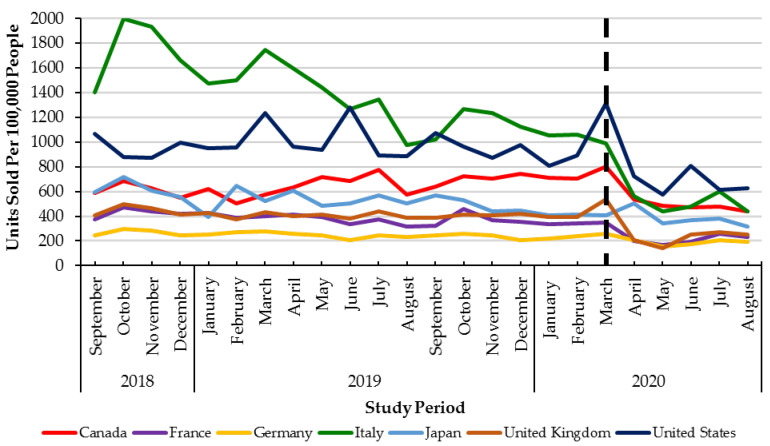
Direct Acting Antiviral units sold per 100,000 people from September 2018–August 2020 across the G7 countries. Dashed lined: declaration of the COVID-19 pandemic (March 2020).

Monthly utilization of DAAs was stable across G7 countries between September 2018 to February 2020 (pre-healthcare restrictions) (Figure 2). In the early months of the pandemic (March to August 2020), all countries in the G7 experienced declines in monthly DAA utilization ranging from −58% (Italy) to −18% (Canada). However, only four countries in the G7 (Canada, Germany, the United Kingdom, and the United States of America) had statistically significant (*p* < 0.05) declines in utilization (Table 1, Figure 2). 

## 4. Discussion

Our study, using global prescription data representing 5.4 billion people from 54 countries, shows a decline in DAA utilization associated with the COVID-19 pandemic across the majority of countries studied (46 out of 54). Importantly, all high-prevalence (HCV prevalence > 2%) countries in the database experienced declines in utilization. Across the major developed economies (the G7), we also observed a decreasing trend in DAA utilization during the early months of the pandemic that continued to remain below pre-pandemic levels by August 2020. These results highlight the impact of the pandemic to the management of HCV and may have set back the ability to achieve the WHO goal of eliminating HCV by 2030. In addition, this slowdown in HCV treatment may lead to an increased number of HCV related comorbidities during this time. 

The COVID-19 pandemic has placed significant strain on national healthcare systems [22]. Although early responses to the pandemic varied across jurisdictions, the redeployment of healthcare resources and public health personnel to focus on containing the COVID-19 pandemic was common. The ensuing healthcare disruptions are known to have had far-reaching consequences for the management of patients with chronic disease, including HCV [23,24]. For example, in February 2020, the Italian government planned to conduct birth cohort screening for hepatitis; however, these programs were halted because of the early exposure to COVID-19 [25]. Similarly, in Egypt, all hepatitis screening programs were paused in March 2020 and the number of HCV treatment units operating was reduced by more than 75% [25]. Our findings build upon these early observations to provide the first estimate of a change in DAA utilization associated with the COVID-19 pandemic on a global scale. 

Our results are concerning as they highlight that the pandemic may be a major speed bump on the WHO’s goal of eliminating HCV by 2030. This plan targets an 80% reduction in new chronic infections and a 65% reduction in mortality from 2015 levels [8]. Of the 45 developed countries, only 11 (Australia, Canada, France, Germany, Iceland, Italy, Japan, Spain, Sweden, Switzerland, and the United Kingdom) were on track to meet elimination targets by 2030 [26]. A further five countries (Austria, Malta, the Netherlands, New Zealand, and South Korea) were on track to meet the targets by 2040 [26]. Interestingly, all the countries listed above that are available in our database (Iceland and Malta not in database) experienced reductions in DAA utilization. This suggests that, even prior to COVID-19, many developed countries were behind schedule to reach the elimination targets. The COVID-19 associated reductions in DAA treatment observed in our study are likely to exacerbate already delayed plans for HCV elimination. Secondly, to receive access to HCV treatment, most countries require patients to receive HCV lab testing and screening [27]. However, public health initiatives (e.g., testing and screening) have fallen behind in both developed and developing nations and need to be accounted for in future healthcare planning and re-launch strategies. This problem is of exceptional concern among the most marginalized populations in developed countries (e.g., people who inject drugs), as well as developing countries with high HCV prevalence and whereby HCV infrastructure and systems may not be as robust to make up for lost opportunities and time [28,29,30]. Consequently, restoring and ramping up HCV screening and treatment program capacity is urgently needed to mitigate the impact of COVID-19 on attaining elimination targets and optimizing the health of patients with HCV. Future work should monitor drug utilization trends from September 2020 onwards, combined with outcome information to more carefully evaluate the impact of the COVID-19 pandemic on HCV elimination goals.

Our study has some limitations. First, the IQVIA data source only includes aggregate DAA units sold without clinical information and patient information (i.e., age, sex). Future work should examine the number of patients impacted across jurisdictions. The results presented in this study align with a previous analysis using patient-level data that observed a sharp and significant decline (−49%) in the number of DAA recipients upon the introduction of COVID-19 restrictions in the first quarter of 2020 in Ontario, Canada [24]. Second, the numbers reported by MIDAS are approximations based on wholesale reports, which may not accurately reflect DAA utilization in every country. For some countries, the percent change (2020 vs. 2019) may be understated as these countries could have bulk ordered DAAs in March 2020 due to fear of drug shortages. Interestingly, few countries exhibited growth, this observation may potentially be associated with signs of bulk ordering, lower baseline rates of treatment that increased prior to the pandemic, or increasing public health initiatives just prior to the pandemic. Lastly, direct comparisons between countries should be contextualized within jurisdictional differences in healthcare system structures and drug distribution. There are important variations in DAA utilization between countries before and during the pandemic; therefore, the impact of COVID-19 in each country should be interpreted within the context of their pharmaceutical policies. For example, DAA utilization in Italy was declining in the year prior to the pandemic, whereas other G7 countries appeared to have stable utilization in 2019.

## 5. Conclusions

The systematic response to COVID-19 led to a large decrease in DAA utilization globally. Delays in starting DAAs and interruptions in treatment places patients with HCV at risk of poor outcomes. Deliberate efforts to counteract the impact of COVID-19 on treatment delivery are needed to support the goal of HCV elimination.

## Figures and Tables

**Table 1:** Results of interrupted time-series model examining the impact of the COVID-19 pandemic on Direct Acting. Antiviral utilization across the G7 countries.

G7 Countries	Percent Change ^1^ (%)	Change in Trend of Monthly Direct Acting Antiviral Utilization ^2^
Canada	−18	*p* = 0.0299
France	−37	*p* = 0.3399
Germany	−19	*p* = 0.0150
Italy	−58	*p* = 0.2008
Japan	−28	*p* = 0.7015
United Kingdom	−32	*p* = 0.0004
United States of America	−24	*p* = 0.0003

^1^*Percent change calculation*. March 2020–August 2020 compared to March 2019–August 2019. ^2^*Time series analysis*. Ramp from April 2020 to August 2020 (September 2018 to March 2020 compared to April 2020 to August 2020).

## Supplementary Materials

The following are available online at https://www.mdpi.com/article/10.3390/v13071314/s1, Table S1: List of Direct Acting Antivirals. Table S2: Proportion of total pharmacy sales obtained by IQVIA-MIDAS database, stratified by country.
