# Supplementary material for: Global Utilization Trends of Direct Acting Antivirals (DAAs) during the COVID-19 Pandemic: A Time Series Analysis
Source: Viruses. 2021 Jul 7;13(7):1314. doi: 10.3390/v13071314 (PMC8310258; doi:10.3390/v13071314)
Supplement: Supplementary file 1 [file viruses-13-01314-s001.zip › viruses-1196664 sa proofa suppl.pdf]

viruses MDPI

Table S1. List of Direct Acting Antivirals.

Direct Acting Antiviral (DAA) Drug List	
**Name**	**Anatomical Therapeutic Chemical (ATC) Code**
Boceprevir	J05AP03
daclatasvir	J05AP07
Dasabuvir	J05AP09
Dasabuvir, Ombitasvir, Paritaprevir and Ritonavir	J05AP52
Elbasvir	J05AP10
Elbasvir and Grazoprevir	J05AP54
Glecaprevir and Pibrentasvir	J05AP57
Grazoprevir	J05AP11
Ombitasvir, Paritaprevir and Ritonavir	J05AP53
Ribavirin	J05AP01
Simeprevir	J05AP05
Sofosbuvir	J05AP08
Sofosbuvir and Ledipasvir	J05AP51
Sofosbuvir and Velpatasvir	J05AP55
Sofosbuvir, Velpatasvir and Voxilaprevir	J05AP56
Telaprevir	J05AP02

Table S2. Proportion of total pharmacy sales obtained by IQVIA-MIDAS database, stratified by country.

Country	Market Covered by MIDAS (%)
Argentina	73
Australia	97
Austria	100
Belarus	100
Belgium	99
Bosnia	95
Brazil	97
Bulgaria	98
Canada	100
Central America	Missing
Chile	71
China	72
Czech Republic	95
Denmark	100
Egypt	75
Estonia	88
Finland	100
France	100
French West Africa	86

Germany	100
Hungary	100
India	95
Ireland	100
Italy	100
Japan	100
Kazakhstan	100
Korea	99
Latvia	100
Lituania	99
Mexico	100
Morocco	88
Netherlands	Missing
New Zealand	97
Norway	100
Pakistan	85
Poland	100
Portugal	100
Puerto Rico	Missinng
Romania	100
Russian Federation	98
Serbia	93
Slovakia	97
Slovenia	98
South Africa	100
Spain	99
Sweden	100
Switzerland	100
Taiwan	97
Thailand	93
Turkey	100
United Arab Emirates (UAE)	45
United Kingdom (UK)	89
